# Two ways to improve myoelectric control for a transhumeral amputee after targeted muscle reinnervation: a case study

**DOI:** 10.1186/s12984-018-0376-9

**Published:** 2018-05-10

**Authors:** Yang Xu, Dingguo Zhang, Yang Wang, Juntao Feng, Wendong Xu

**Affiliations:** 1 State Key Laboratory of Mechanical System and Vibration, School of Mechanical Engineering, Shanghai Jiao Tong University, Dongchuan Road, Shanghai, 200240 China; 2 Department of Hand Surgery, Huashan Hospital, Fudan University, Wulumuqi Road, Shanghai, 200040 China

**Keywords:** Surface electromyography, Targeted muscle reinnervation, Rehabilitation training, Myoelectric control, Pattern recognition

## Abstract

**Background:**

Myoelectric control of multifunctional prostheses is challenging for individuals with high-level amputations due to insufficient surface electromyography (sEMG) signals. A surgical technique called targeted muscle reinnervation (TMR) has achieved impressive improvements in myoelectric control by providing more sEMG control signals. In this case, some channels of sEMG signals are coupled after TMR, which limits the performance of conventional amplitude-based control for upper-limb prostheses.

**Methods:**

In this paper, two different ways (training and algorithms) were attempted to solve the problem in a transhumeral amputee after TMR. Firstly, effect of rehabilitation training on generating independent sEMG signals was investigated. The results indicated that some sEMG signals recorded were still coupled over the targeted muscles after rehabilitation training for about two months. Secondly, pattern recognition (PR) algorithm was then applied to classify the sEMG signals. In the second way, to further improve the real-time performance of prosthetic control, a post-processing method named as mean absolute value-based (MAV-based) threshold switches was utilized.

**Results:**

Using the improved algorithms, substantial improvement was shown in a subset of the modified Action Research Arm Test (ARAT). Compared with common PR control without post-processing method, the total scores increased more than 18% with majority vote and more than 58% with MAV-based threshold switches. The amputee was able to finish all the tasks within the allotted time with the standard MAV-based threshold switches. Subjectively the amputee preferred the PR control with MAV-based threshold switches and reported it to be more accurate and much smoother both in experiment and practical use.

**Conclusions:**

Although the sEMG signals were still coupled after rehabilitation training on the TMR patient, the online performance of the prosthetic operation was improved through application of PR control with combination of the MAV-based threshold switches.

**Trial registration:**

Retrospectively registered http://www.chictr.org.cn/showproj.aspx?proj=22058.

**Electronic supplementary material:**

The online version of this article (10.1186/s12984-018-0376-9) contains supplementary material, which is available to authorized users.

## Background

Myoelectric upper-limb prostheses where surface electromyography (sEMG) signals are utilized as control sources have been developed for a long time [1, 2]. However, control of a prosthesis with multiple degrees of freedom (DoFs) including elbow, wrist and hand for high-level amputees is still difficult due to lack of sufficient sEMG signals. In the conventional control, users can only operate one joint at a time using a residual pair of agonist-antagonist muscles [3]. They have to sequentially operate one joint of the prostheses with a “mode switch” [4]. This type of myoelectric control is not intuitive because users are not able to control the prostheses naturally, making the operation cumbersome and frustrating [5].

Regarding high-level amputation, the neural information for the limb motion is still available in the brachial plexus nerves. Therefore, targeted muscle reinnervation (TMR) has been developed to provide rich sources of physiologically appropriate motor control inputs for controlling multiple-DoF upper-limb prostheses [6]. With TMR, the residual nerves in the amputated limb are transferred to spare muscles, e.g. the pectoralis major muscles [6, 7]. The targeted muscles are denervated first in the surgery, and then residual brachial plexus nerves are anastomosed to them. These reinnervated muscles will later serve as biological amplifiers of the transferred nerve signals. After recovery, more channels of independent sEMG signals, which derive from residual nerves, can be recorded over the regions of targeted muscles. It can contribute to improving the control of prostheses with multiple DoFs. In addition, the users may feel more natural in the prostheses operation. The recorded sEMG signals used to control the motion in the prostheses are similar to that in the natural arm. Prior research has already demonstrated TMR to be a valid method for upper-limb prostheses control [4, 6–11].

The conventional myoelectric control of prostheses in TMR is an amplitude-based control method [12]. Mean absolute value (MAV) of the sEMG signals recorded is often used to compare with the preset threshold to determine which kind of motion the user intends to do. The amplitude-based method is simple and effective. Also, it becomes possible to simultaneously actuate multiple joints at a time if independent sEMG signals can be acquired. However, it may be hard to ensure the isolation of sEMG signals all the time after TMR which will lead to the failure of amplitude-based control.

Regarding a full human-machine interface, both sides, involving “human” (user) and “machine” (algorithm), should be brought into focus in order to improve the performance of prosthetic control. Previous research has demonstrated that the learning ability of users makes a difference in the operation of prostheses. Mutual adaptation between subjects and adaptable prosthetic hand was investigated in a prosthetic hand system [13]. Powell et al. found performance of prosthetic control could be improved after two weeks’ training with visual biofeedback [14]. Even without external feedback, amputees could adapt to the way of muscle contraction after training [15]. Hahne et al. verified that man-machine co-adaptation was helpful with better myoelectric control [16]. Training strategy of providing clustering-feedback was proposed to help adjust subjects’ motion gestures and forces of muscle contraction [17]. Although the subject could have learned to activate the nerve-muscle unit independently through enough practice, sEMG of the targeted channel might not be decoupled from the other channels. To our best knowledge, no experiment has been done to demonstrate whether it is possible for amputees with coupled sEMG after TMR to learn to activate the targeted muscles independently with long-term rehabilitation training.

Pattern recognition (PR) algorithm is a well-recognized method to control a dexterous prosthesis. It is well known that the independence of sEMG signals is not a must in PR control [18]. Different kinds of features, such as time-domain [5, 19], frequency-domain [20] and combined time-frequency features [21, 22], were utilized in order to improve the performance of prosthetic control. Apart from these, classification performance of various classifiers was investigated, including linear discriminant analysis (LDA) [23], support vector machines [24], neural networks [25] and hidden Markov models [26]. Notably, for the sake of lower error rate in real-time control, several post-processing methods like majority vote [23] and a decision-based velocity ramp [27] were proposed. PR control re-attracted some attention recently, which was compared with conventional amplitude-based control in TMR amputees [12]. Other studies demonstrated that more myoelectric control commands could be elicited through PR rather than just limited by the targeted sites [28, 29]. The effectiveness of nontargeted-electrode placement [18] and reduced-electrode placement in PR [30] was also investigated.

In this case, we found the problem that some coupled sEMG was recorded from a transhumeral amputee after TMR. We aimed to solve this problem and improve myoelectric control from two perspectives. Rehabilitation training was first conducted to assess how rehabilitation training influenced the independence of sEMG signals measured over the targeted muscles and if the subject would learn to activate the targeted muscles independently with ease. A PR-based algorithm was then applied to process the sEMG signals due to the unsatisfying result of rehabilitation training. To further improve the performance of real-time prosthetic control, MAV-based threshold switches were utilized as the post-processing method. The results of functional testing indicated effectiveness of the improved PR control. The subject also preferred the improved PR control inpractical use.

## Methods

### Surgery and recovery

**Fig. 1:**
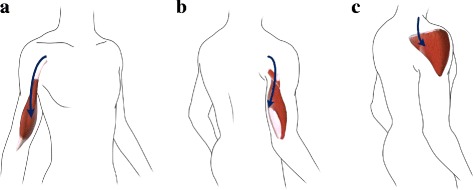
Diagram of TMR surgery in the subject with transhumeral amputation. **a** The median nerve was transferred to the medial head of the biceps. **b** The distal part of the radial nerve was sewn on to the long head of triceps branch. **c** The ulnar nerve was anastomosed to anterior part of the deltoid branch

This study got the approval from the Ethics Committee of Shanghai Huashan Hospital. The subject is a 45-year-old male who once suffered from traumatic right upper limb injury, and a transhumeral amputation was performed on him. About six months after the amputation, TMR surgery was performed on him at Department of Hand Surgery in Huashan Hospital with the informed consent from him. The median nerve in the impaired side was transferred to the branch of the musculocutaneous nerve innervating the short head of the biceps. Similarly, the distal part of the radial nerve was sewn on to the long head of triceps branch and the ulnar nerve was anastomosed to anterior part of the deltoid branch. Subcutaneous fat tissues over interior biceps and lateral triceps were removed to enhance the quality of sEMG signals. The subcutaneous fat saved was then tucked down between the short head and long head of biceps, and the same on the triceps to prevent sEMG cross-talk as much as possible. The short head of biceps and the long head of triceps were intended for control of hand close and open. The anterior part of deltoid was used for control of wrist rotation in one direction. The long head of the biceps and medial head of the triceps were preserved to control the elbow flexion and extension. The diagrammatic sketch of TMR surgery in the subject with transhumeral amputation is shown in Fig. 1.

No complication occurred after the subject recovered from surgery. To promote the reinnervation of targeted muscles, the subject was instructed to frequently attempt to open and close the right hand and abduct the right hand fingers in his daily life. It was helpful for the activation of short head of the biceps, long head of triceps and anterior part of the deltoid. After approximately 3 months, all the targeted muscles had been reinnervated. When the subject tried to close and open his right hand, obvious contraction could be observed and strong sEMG signals could be recorded over the short head of the biceps and long head of triceps. The right hand finger’s abduction caused a mild contraction of the anterior part of the deltoid. sEMG signals could also be recorded, but the amplitude was lower than that recorded over the short head of the biceps and long head of triceps. Although anterior part of deltoid was activated through abduction of right hand fingers after TMR surgery, the sEMG signals over the location were used for wrist rotation. In this paper that follows, wrist rotation refers to motion attempt of abducting right hand fingers. The subject was also asked to rotate the wrist of right hand instead of abducting right hand fingers in daily practice.

### Rehabilitation training

Although sEMG signals could be recorded from all the five targeted regions of muscles, it was noticed that the subject was not able to activate the targeted muscle corresponding to the intended motion independently, i.e. sEMG signals were recorded simultaneously from both targeted and nontargeted muscles. For example, when the subject intended to open his right hand, apart from the activation of the long head of triceps, sEMG signals could also be recorded over other targeted muscles. More notably, when the subject tried to open his right hand, the amplitude of sEMG signals over medial head of the triceps, which was used for control of elbow extension, was almost same with that when the subject actually intended to extend his elbow. Similar phenomena occurred when the subject tried to activate other targeted muscles, which made the direct amplitude-based EMG control invalid. The subject reported that he just performed the single motion attempt as required while multiple sEMG signals could be recorded.

**Fig. 2:**
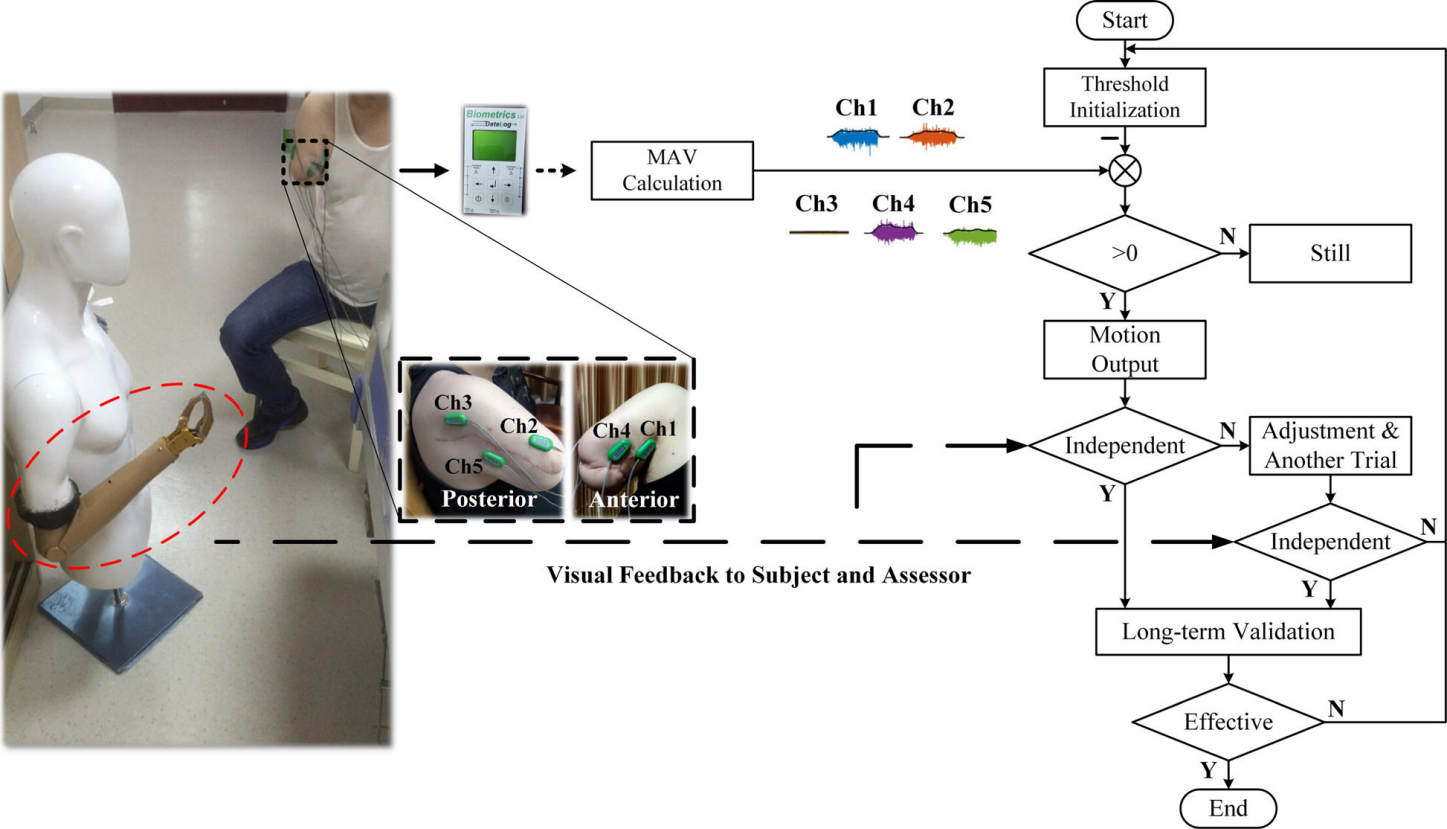
The scheme of rehabilitation training. sEMG signals were recorded by five bipolar electrodes located on the targeted muscles and sent from DataLog (Biometrics Ltd, USA) to computer through bluetooth. MAV of each channel was then calculated and corresponding motion command determined by the training protocol was sent to the prosthesis. The movement of prosthesis could been seen by the subject and assessor. The local enlarged drawing shows the electrode configuration

The rehabilitation training guided by a therapist was conducted to help the subject learn to activate the targeted muscles independently. Figure 2 displays the scheme of rehabilitation training. A commercial prosthesis (Danyang Prostheses Co. Ltd, China), the embedded control system of which was developed by ourselves, mounted on a model was put in front of the subject at a distance of about 1 m. At the beginning of training, threshold of each motion was preset based on the corresponding MAV of recorded sEMG signals elicited by a moderate level of contraction. As long as the MAV of sEMG signals was higher than the threshold of that channel, the corresponding motion command would be sent to the control system of prosthesis and the joint would then start to move. The EMG channels 1 to 5 corresponded to hand close, hand open, wrist rotation, elbow flexion and elbow extension, and there was a preset threshold for each one. Specifically, if the subject was told to close his hand, the MAV of channel 1 (Ch1) was calculated and compared with the preset threshold for CH1. If the MAV was higher than the preset threshold of CH1, the prosthesis generated the motion of hand close. Otherwise, the prosthesis kept still. In the rehabilitation, the subject was told to try to close/open his right hand, rotate his right hand wrist and flex/extend his right elbow in each session. The order of the motions mentioned above was randomized. In the process, an occupational therapist noted each motion of the prosthesis, which of them finished independently and which of them finished with other unintended movement. The subject himself could also see the motions of the prosthesis. For the latter case, the subject would be asked to carefully imagine how to produce the corresponding motion physiologically and try to adjust his way of motion attempt, the purpose of which was to generate the motion independently. If it did not work, the threshold preset would be modified to be more close to the independent output of intended motion. The subject was then required to finish another complete session again to check the effectiveness of previous modified threshold. If each motion was performed independently, the threshold was preserved to check whether it would be valid in the following longterm use. If not, the threshold would be modified again. The rehabilitation training, duration of which was approximately 2 h per time, was done iteratively like the above procedure four times per week. It lasted for about two months in the hospital. The sEMG signals of each motion were recorded once a week to assess the influence of training. The subject went back home and had a rest at weekends.

### sEMG signal recording and processing

During rehabilitation training, the sEMG signals were sampled at 1000 Hz with a bandwidth of 20-460 Hz and a gain of 1000 using Biometrics system (Biometrics Ltd, USA). The targeted skin was first cleaned with alcohol. Five channels of bipolar electrodes were then attached to the skin by double sided adhesive tape over the reinnervated short head of biceps (Ch1), long head of triceps branch (Channel 2, Ch2) and anterior part of the deltoid branch (Channel 3, Ch3), along with long head of the biceps (Channel 4, Ch4) and medial head of the triceps (Channel 5, Ch5). A reference electrode was located on the bone area of left wrist. The electrode configuration was determined first according to the clinical experience under the guidance of a doctor and marked on the subject’s skin then (Fig. 2). To assess the influence of rehabilitation training, sEMG signal were measured on the subject once a week when he performed 10 sessions of required movements, each of which consisted of closing his right hand, opening his right hand, rotating his right hand wrist, flexing his right elbow and extending his right elbow following a video demonstration. Each movement was required to hold for about 5 s at a moderate level of effort. Average MAV of each motion was then calculated based on the 10 sessions for further analysis. To avoid muscle fatigue, the subject could have a rest for 10s between each movement and about two minutes between each session.

For the real-time control of prosthesis, sEMG signals were filtered first with a bandwidth of 80-400 Hz and amplified with a gain of 10000 using the electrodes made by Danyang Prostheses Co. Ltd. Signals were then sampled at 1860 Hz with the ADC module of embedded system. The electrode configuration was same with that during rehabilitation training except that the reference electrode was not used.

The analysis window length of PR algorithm was determined to be 137.6 ms with 50% overlap. Four time domain features were utilized for classification of the intended movements, which included MAV, waveform length, number of zero crossings and number of slope sign changes. A linear discriminant analysis (LDA) classifier based on Bayesian decision was created with the features extracted from the training set [31]. The LDA classifier could then be used for the real-time control of the prosthesis. After the subject put on the prosthesis, a training session was firstly needed to obtain the parameters of the LDA classifier for the following realtime operation. In order to prompt the subject which motion he should do at the right time in the training session, the prosthesis would produce the real movement of each joint. Our subject was required to do the same motion as the prosthesis did and held during that sampling time. The total time of training session was about 35 s, sampling time of each motion was about 7 s. The first 4 s was cut to avoid the effect of transient signals and only the last 3 s was utilized for training of the classifier.

### Post-processing

The transient signals were not utilized to train the LDA classifier, so the recognition accuracy can be low when the subject switched from one motion to another. This really frustrated the subject because he thought the prosthesis was out of control. The MAV of each channel was then taken into consideration to discriminate between transient and steady state. Principle of the post-processing method is quite simple. Final output of motion *M*_*i*_ is determined according to

$$M_{i}=C_{i}{\textcircled{+}}D_{i} $$

where *i* is the class of motion and *C*_*i*_ is the classified motion with no post-processing within current analysis window. + indicates that if *D*_*i*_>0, final output of motion *M*_*i*_ will equal to *C*_*i*_. Otherwise, the prosthesis will be still. More specifically, only when a motion is classified by PR and the MAV of the sEMG signals from classified motion’s channel is higher than the preset threshold will the prosthesis output that motion. For example, if the output of the PR is hand close and the MAV of Ch1 is higher than the pre-set threshold, then the prosthetic hand will start to close. *D*_*i*_ is the difference between the MAV of the classified motion *V*_*i*_ and the preset threshold of classified motion *T*_*i*_. Its definition can be seen as follows

$$D_{i}=V_{i}-T_{i} $$

**Fig. 3:**
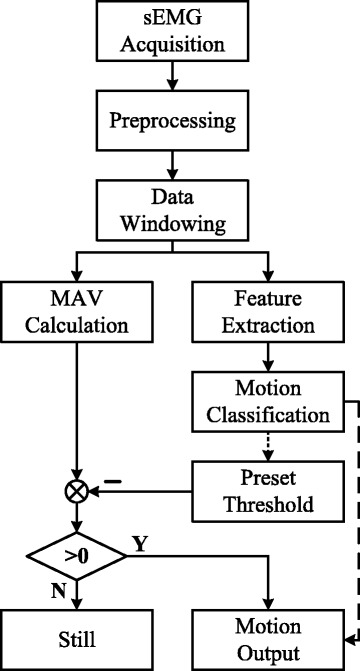
Flowchart of the Improved PR with MAV-based threshold switches. Only when a motion is classified by PR and the MAV of the sEMG signals from classified motion’s channel is higher than the preset threshold will the prosthesis output that motion

MAV can be effectively applied to discriminate between the transient and steady states. In practical use, *T*_*i*_ is preset manually based on the real-time performance of prosthetic control. The MAV-based threshold switches eliminated the adverse effect of transient signals to a great extent. This may bring a little time delay in that the transient-state motions would be recognized as staying still. However, the subject thought it acceptable and better result of functional testing can be achieved. The flowchart of the improved PR is shown in Fig. 3.

### Functional testing

Comparisons were made between common PR algorithm with no post-processing (Control), majority vote and MAV-based threshold switches using a subset of the modified Action Research Arm Test (ARAT). Three different lengths of majority vote queue were selected as 3 (L3), 5 (L5) and 10 (L10) [23]. Three different thresholds were also preset. Standard threshold (ST) was defined as 0.2 mV (Ch1), 0.2 mV (Ch2), 0.1 mV (Ch3), 0.2 mV (Ch4) and 0.2 mV (Ch5), which worked well in practical use. Lower threshold (LT) was determined as 80% of ST and higher threshold (HT) was determined as 120% of ST. To minimize the effect of muscle fatigue, whole experiment was divided into following 7 conditions in the order of Control, L10, ST, LT, HT, L3 and L5. The subject was required to finish 8 tasks selected from ARAT for each condition and each task should be repeated for 3 times. When the algorithm was changed, the subject was allowed to have a rest and then practice each task for several times.

ARAT is considered to be appropriate for assessing recovery of upper limb function [32, 33]. All the six items in grasp subscale except moving the biggest block (10 cm × 10 cm × 10 cm) and three items including pouring water from one glass to another, displacing alloy tubes (2.25 cm, 1 cm) from one side of table to the other in grip subscale were selected to make up of the whole functional testing, which was almost consistent with [34]. The general principle of performing and scoring ARAT referred to [32, 33] with slight modifications. The subject wore the prosthesis (Danyang Prostheses Co. Ltd, China) in the functional testing. Instead of sitting upright on a chair, the subject started the tests standing with both left hand and right prosthesis naturally down in front of the table at a distance of about 30 cm.

## Results

### Surgery

In this case, all the three nerve-muscle units which utilized median nerve, distal radial nerve and ulnar nerve, were successfully obtained after TMR surgery. With the long head of the biceps and medial head of the triceps taken into consideration, sEMG signals could be recorded over all the five targeted muscles. A problem was also noticed that independent sEMG signals could not be recorded in which condition the conventional amplitude-based control was invalid.

### Rehabilitation training

**Fig. 4:**
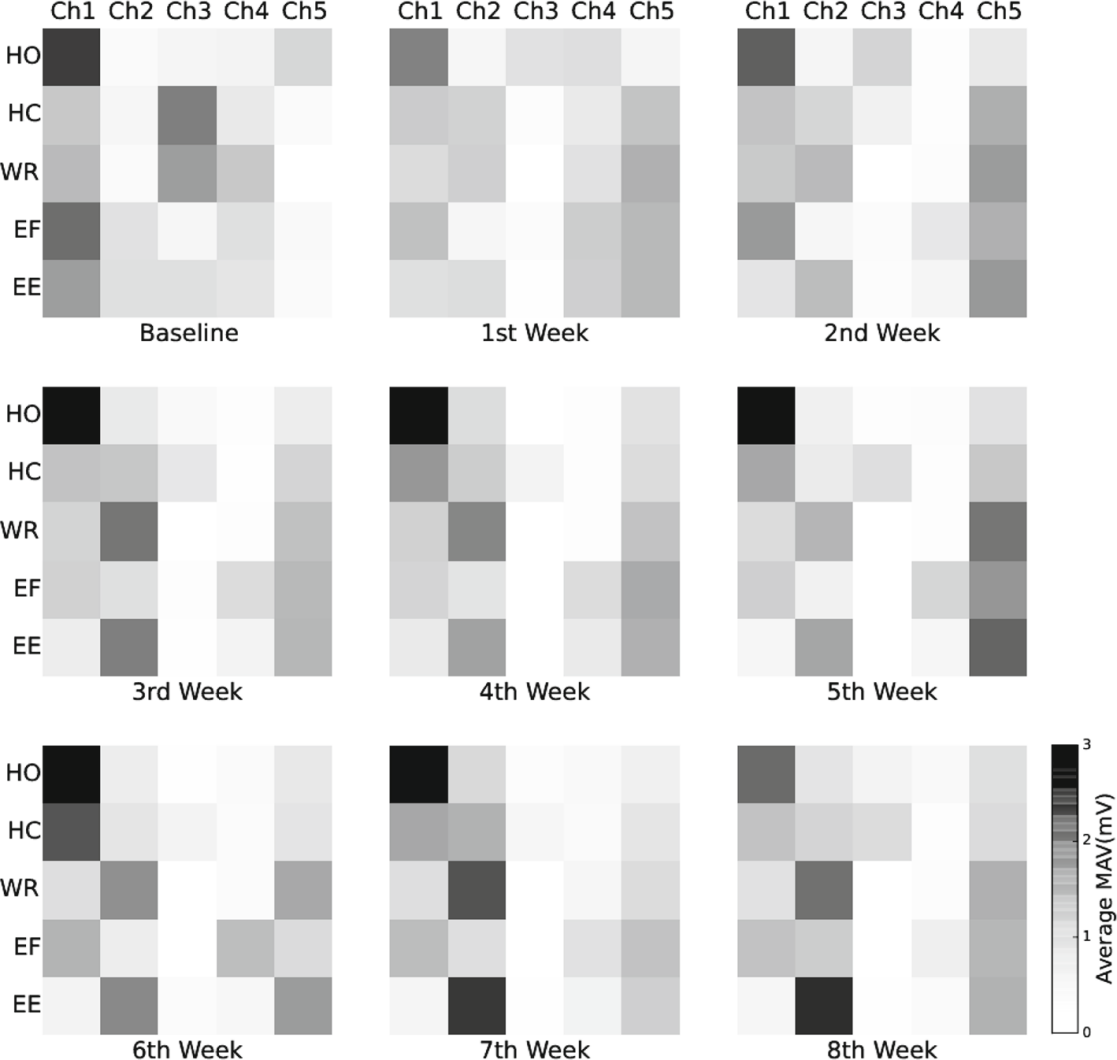
Changes of the subject’s sEMG signals recorded every week. HO, HC, WR, EF and EE represents hand open, hand close, wrist rotation, elbow flexion and elbow extension respectively. Average MAV of sEMG signals from each channel is represented in a 5 ×5 grey scale block. The first block displays the average MAV of sEMG signals recorded before rehabilitation. The first row of each block shows the average MAV of each channel when the subject intended to close his hand. Similarly, the second to fifth row of the block respectively represents the hand open, wrist rotation, elbow flexion and elbow extension

The aim of rehabilitation training was to investigate if independent sEMG signals could be obtained so that amplitude-based control could be directly applied. Changes of the subject’s sEMG signals recorded every week during rehabilitation training are shown in Fig. 4. The average MAV on the principle diagonal should ideally be the biggest compared with that in each corresponding column. It means that the MAV of right motion user intends is bigger than that when the user intends to do other motions and then the simple amplitude-based control will be effective. Figure 4 shows that the average MAV of hand close and elbow extension became stronger with rehabilitation training while the average MAV of hand open, wrist rotation and elbow flexion did not change significantly. No obvious improvement with the independence of sEMG signals can be seen from the series of blocks.

### Functional testing

The results of whole experiment are summarized in Table 1. The time of each task cost is listed in the table and the completion rate is calculated. The ARAT scores of all conduced tasks are also provided (Additional file 1: ARAT scores).

**Table 1:** Time of each task cost in functional testing ARAT

Items of ARAT	No.	Control	L3	L5	L10	LT	ST	HT
Block 2.5 cm ×2.5 cm ×2.5 cm	1	18.7	11.3	-	13.9	24.6	35.6	11.7
	2	- ^a^	- -	-	21.0	10.6	26.5	15.9
	3	- - ^b^	14.0	53.6	32.0	15.3	13.0	14.8
Completion rate (%)		33	67	33	100	100	100	100
Block 5.0 cm ×5.0 cm ×5.0 cm	1	18.4	8.5	14.4	26.3	11.1	11.2	22.5
	2	- -	16.5	11.4	28.5	10.7	10.5	40.2
	3	17.4	11.6	19.9	- -	20.9	10.2	21.4
Completion rate (%)		67	100	100	67	100	100	100
Block 7.5 cm ×7.5 cm ×7.5 cm	1	-	- -	-	9.7	23.4	35.8	38.7
	2	-	-	-	8.3	11.0	19.8	15.2
	3	-	-	-	7.5	11.4	35.6	23.1
Completion rate (%)		0	0	0	100	100	100	100
Ball diameter 7.5 cm	1	- -	-	11.9	8.4	12.0	11.6	11.2
	2	-	10.0	- -	8.8	10.8	11.3	12.0
	3	- -	41.7	18.8	8.5	7.2	14.1	9.7
Completion rate (%)		0	67	67	100	100	100	100
Stone 10.0 cm ×2.5 cm ×1.0 cm	1	-	19.1	15.5	15.4	11.6	11.7	11.6
	2	11.7	15.6	15.0	12.2	6.3	12.3	9.9
	3	44.1	-	-	10.9	10.0	13.6	9.2
Completion rate (%)		67	67	67	100	100	100	100
Pouring water	1	- -	- -	21.7	- -	22.2	27.5	- -
	2	- -	- -	- -	- -	17.8	18.4	- -
	3	- -	- -	- -	- -	- -	15.7	19.9
Completion rate (%)		0	0	33	0	67	100	33
Tube 2.5 cm ×16.0 cm	1	57.6	- -	- -	- -	16.6	12.6	10.8
	2	-	- -	11.4	- -	19.9	19.2	12.0
	3	- -	- -	- -	11.6	-	14.1	10.8
Completion rate (%)		33	0	33	33	67	100	100
Tube 1.0 cm ×16.0 cm	1	- -	- -	- -	- -	- -	44.7	- -
	2	- -	- -	- -	- -	- -	17.2	- -
	3	- -	- -	- -	- -	26.1	15.6	21.9
Completion rate (%)		0	0	0	0	33	100	33

^a^The subject could not finish the trials within 1 min [30]

^b^The subject failed the task, e.g. he touched the object with the nontested hand

**Fig. 5:**
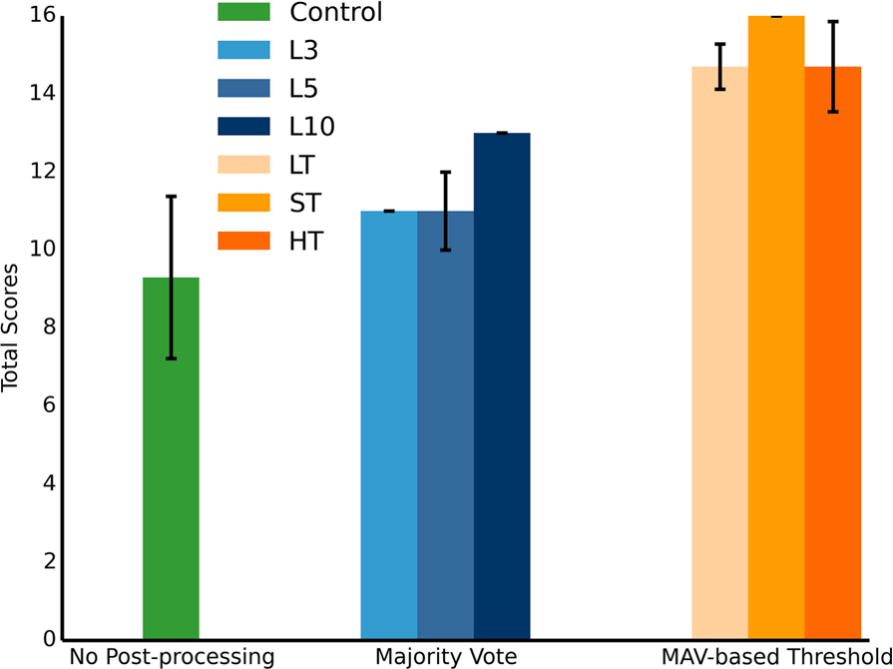
Total scores of each condition with different post-processing methods in ARAT. Final score of each task is the average of all three trials. In the group of majority vote, three different lengths of majority vote queue were selected as 3 (L3), 5 (L5) and 10 (L10). In the group of MAV-based threshold, standard threshold (ST) was defined as 0.2mV (Ch1), 0.2mV (Ch2), 0.1mV (Ch3), 0.2mV (Ch4) and 0.2mV (Ch5), which worked well in practical use. Lower threshold (LT) was determined as 80% of ST and higher threshold (HT) was determined as 120% of ST

Figure 5 shows the total score of each condition with different post-processing method in the modified ARAT. The final score of each task is the average of all three trials. The final score of common PR control without post-processing method is 9.3. The final score of PR control is 11 (L3), 11 (L5), 13 (L10) with majority vote and 14.7 (LT), 16 (ST), 14.7 (HT) with MAV-based threshold switches.

**Fig. 6:**
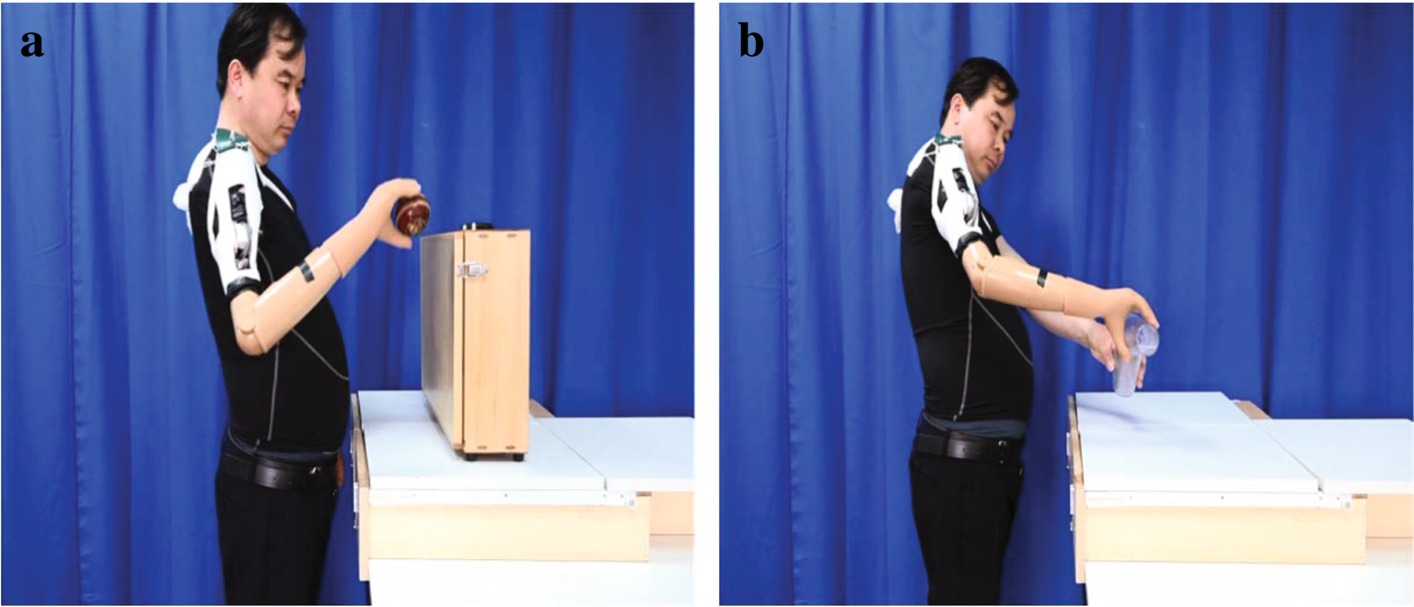
The subject performing ARAT using MAV-based threshold switches. **a** The subject was moving the ball onto the shelf. **b** The subject was pouring water from one glass to another

Subjectively the subject reported that he was satisfied with the improved PR control with MAV-based threshold switches for the accurate output of intended motion. The subject felt that he fully possessed control of the prosthesis. Despite a little time delay, the results of objective functional testing indicated the effectiveness of MAV-based threshold switches. Figure 6 displays the subject performing the modified ARAT using improved PR control with MAV-based threshold switches. A video can be seen as well (Additional file 2: Video).

## Discussion

A TMR case in which sEMG signals measured were coupled is reported in this paper. Independent sEMG signals are expected in clinical practice after TMR surgery. Simple amplitude-based control can then be directly used for control of prostheses, which was proposed in the first TMR case [6]. Prior research has demonstrated that the independent sEMG signals could be recorded over the reinnervated muscles in most cases [6–8]. However, sEMG signals vary among different individuals, and the subject with transhumeral amputation in this case encountered the problem of coupled sEMG signals.

The reasons why the sEMG signals recorded from the amputee were coupled in this case may be attributed to the two common factors: cross-talk and co-contraction. Prior research has shown that cross-talk will not be a problem if the distance of selected muscles is more than 2 cm [35]. After careful examination, we found the distance between the two closest electrodes is greater than 2 cm. So cross-talk was not considered to be the main reason for the coupled sEMG in this case. However, the unsatisfying results may be caused by other deeper factors. First of all, the selection of targeted muscles has a great impact on the final quality of sEMG signals. In this subject, the medial and long head of triceps were selected as two muscle units while they are just two different branches of the same muscle. Actually, amplitude of the two channels was almost same in signal recording. As a result, it was hard to distinguish from the two channels just according to MAV. However, risk of surgery should also be taken into consideration, and free selection of targeted muscles is often practically not allowed. It brings the possibility that the coupled sEMG signals are recorded from some of the channels. Apart from this, it remains to be further investigated if the amputee’s corresponding cortical function has changed after amputation. Pascual-Leone pointed out that amputees can lose their motor control abilities as time passes after amputation [36]. It is possible that the amputee is not able to clearly distinguish each motion of the phantom limb in the brain. Furthermore, there may be some correlation between co-contraction and reorganization of cortical function. In addition, since this is our first case of TMR, the coupled sEMG signals might even indicate the failure of surgery itself. Some knacks of TMR surgery may not be well performed. All the reasons above can lead to the result that the available sEMG signals are coupled.

Since TMR technology has been applied in clinical practice in the world, similar cases that sEMG signals are coupled may be encountered in future. This may be a hindrance that prevents the wide application of TMR. Trying to get independent sEMG signals through rehabilitation training is still the first choice. With the independent sEMG, the simple and effective amplitude-based control can be used, and this algorithm does not need much training as PR control in practical use. More significantly, amplitude-based control may allow controlling multiple joints at the same time. This kind of simultaneous control is intuitive as that we move our natural arms of multiple joints. Unfortunately, our rehabilitation training did not generate independent sEMG on the subject, and thus the amplitude-based control cannot be used. Perhaps this case has an inherent problem that cannot be solved by rehabilitation. Also perhaps our rehabilitation training was not perfect. Communications will be conducted to check the problems with the experienced therapists on TMR rehabilitation [10]. Anyway, the result of our rehabilitation training can be a reference for others in TMR research field. Once similar cases occur, it demands careful consideration before long-term rehabilitation training.

Due to the unsatisfying result of rehabilitation training, we turned to solve the problem in terms of algorithms. Actually, application of PR control does not directly solve the problem that sEMG signals are coupled and just gets around that because independent sEMG signals are not a must in PR control. The post-processing method MAV-based threshold switches was utilized to improve the real-time performance of prosthetic control. One advantage of this post-processing method is that each motion has its own particular preset threshold. In other words, there can be five different threshold switches for the five intended motions in our case. Since each targeted muscle is intended for one motion after TMR, particular preset threshold of each motion will be more specific to discriminate between transient and steady state. The ultimate goal of this method is to eliminate the unintended output of motion, especially when in transient state. Reason for the improved performance seems intuitive. Different from offline analysis, the subject will stop the motion immediately when he notices the unintended output during online test in that he may think the way of motion attempt is wrong and another try is needed. The transient signals are not used for training of classifier, so most of the misclassification will be found when in transient state, which means the subject may often stop just after the beginning of motion attempt and then try it again, creating a vicious circle. Adding a MAV-based threshold switch is a solution, which was validated in ARAT. It discards the recognition results with low MAVs so that the prosthesis will stay still instead of acting with unintended output, especially in transient state.

Improvement of the PR control with MAV-based threshold switches was effective in this case. The subject could accomplish all the tasks within the allotted time in ST condition. Compared with common PR control without post-processing method, the total scores increased more than 58% with MAV-based threshold switches. However, the performance of majority vote is not so satisfying as that of MAV-based threshold switches. The total score increased only 18% in L3 and L5 condition in comparison with common PR control without post-processing. Several factors lead to the result: 1) The L3 and L5 condition were selected as the last two to perform the ARAT. Muscle fatigue may have an adverse effect on the performance. The order of L10 condition was second and the performance in that condition was much better. A 40% increase was achieved compared with common PR control, which was almost same with LT and HT condition. 2) The subject was more experienced with the method of MAV-based threshold switches in that he used it in practical prosthetic hand control.

At present, the performance of the prosthetic control is satisfactory in the clinic lab, but there are still some problems for the daily-life use in practice. The first problem is about the placement of the five EMG electrodes. Initially, the electrodes were embedded in the socket. However, we found it difficult in this way to make the electrodes maintain contact with the skin all the time when the subject was operating the prosthesis. Later, we solved the problem by sewing the electrodes in a tight shirt, but it was inconvenient during the practical use. We will continue to try some other effective and proper methods to set the electrodes in the socket. Apart from this, the prosthesis would slide down slowly during the use due to the weight of the prosthesis, which made it hard for the subject to use the prosthesis for a whole day. A more stable and convenient socket should be designed to solve these problems for the subject in daily life, and reducing the weight of the prosthesis might also be a solution.

## Conclusions

In this case, rehabilitation training and PR algorithm were adopted as two ways to solve the problem that no independent sEMG signals could be recorded over the targeted muscles in a subject with transhumeral amputation after TMR. Although the sEMG signals were still coupled after rehabilitation training, application of PR control realized the control of prosthesis. The MAV-based threshold switches were utilized to improve the real-time performance of prosthetic operation.

## Additional files

Additional file 1 ARAT scores. Scores of all the tasks conducted by the subject in the experiment are given in the score sheet. The tasks were selected from ARAT. (PDF 67 kb)

Additional file 2: Video. The video demonstrates the subject performing two tasks: moving the ball onto the shelf and pouring water from one glass to another. (FLV 8910 kb)

## Abbreviations

ARAT: Action research arm test
Ch1-Ch5: Channel 1-Channel 5
DoF: Degree-of-freedom
HT: Higher threshold
LT: Lower threshold
MAV: Mean absolute value
PR: Pattern recognition
sEMG: Surface electromyogram
ST: Standard threshold
TMR: Targeted muscle reinnervation
