# Supplementary material for: Two ways to improve myoelectric control for a transhumeral amputee after targeted muscle reinnervation: a case study
Source: J Neuroeng Rehabil. 2018 May 10;15:37. doi: 10.1186/s12984-018-0376-9 (PMC5946536; doi:10.1186/s12984-018-0376-9)
Supplement: Supplementary file 1 — ARAT scores. Scores of all the tasks conducted by the subject in the experiment are given in the score sheet. The tasks were selected from ARAT. (PDF 67 kb) [file 12984_2018_376_MOESM1_ESM.pdf]

Table 1. Action Research Arm Test Scoring Sheet in Control Condition

Item	Score		
	Trial 1	Trial 2	Trial 3
Block, 2.5cm^3	2	1	1
Block, 5.0cm^3	2	1	2
Block, 7.5cm^3	1	1	1
Cricket ball	1	1	1
Sharpening stone	1	2	2
Pour water from one glass to another	1	0	1
Displace 2.25-cm alloy tube from one side of table to the other	2	0	1
Displace 1-cm alloy tube from one side of table to the other	1	1	1

Table 2. Action Research Arm Test Scoring Sheet in L3 Condition

Item	Score		
	Trial 1	Trial 2	Trial 3
Block, 2.5cm^3	2	1	2
Block, 5.0cm^3	2	2	2
Block, 7.5cm^3	1	1	1
Cricket ball	1	2	2
Sharpening stone	2	2	1
Pour water from one glass to another	1	1	1
Displace 2.25-cm alloy tube from one side of table to the other	1	1	1
Displace 1-cm alloy tube from one side of table to the other	1	1	1

Table 3. Action Research Arm Test Scoring Sheet in L5 Condition

Item	Score		
	Trial 1	Trial 2	Trial 3
Block, 2.5cm^3	1	1	2
Block, 5.0cm^3	2	2	2
Block, 7.5cm^3	1	1	1
Cricket ball	2	0	2
Sharpening stone	2	2	1
Pour water from one glass to another	2	1	1
Displace 2.25-cm alloy tube from one side of table to the other	1	2	1
Displace 1-cm alloy tube from one side of table to the other	1	1	1

Table 4. Action Research Arm Test Scoring Sheet in L10 Condition

Item	Score		
	Trial 1	Trial 2	Trial 3
Block, 2.5cm^3	2	2	2
Block, 5.0cm^3	2	2	1
Block, 7.5cm^3	2	2	2
Cricket ball	2	2	2
Sharpening stone	1	1	1
Pour water from one glass to another	1	1	1
Displace 2.25-cm alloy tube from one side of table to the other	1	1	2
Displace 1-cm alloy tube from one side of table to the other	1	1	1

Table 5. Action Research Arm Test Scoring Sheet in LT Condition

Item	Score		
	Trial 1	Trial 2	Trial 3
Block, 2.5cm^3	2	2	2
Block, 5.0cm^3	2	2	1
Block, 7.5cm^3	2	2	2
Cricket ball	2	2	2
Sharpening stone	2	2	2
Pour water from one glass to another	2	2	1
Displace 2.25-cm alloy tube from one side of table to the other	2	2	1
Displace 1-cm alloy tube from one side of table to the other	1	1	2

Table 6. Action Research Arm Test Scoring Sheet in MT Condition

Item	Score		
	Trial 1	Trial 2	Trial 3
Block, 2.5cm^3	2	2	2
Block, 5.0cm^3	2	2	2
Block, 7.5cm^3	2	2	2
Cricket ball	2	2	2
Sharpening stone	2	2	2
Pour water from one glass to another	2	2	2
Displace 2.25-cm alloy tube from one side of table to the other	2	2	2
Displace 1-cm alloy tube from one side of table to the other	2	2	2

Table 7. Action Research Arm Test Scoring Sheet in HT Condition

Item	Score		
	Trial 1	Trial 2	Trial 3
Block, $2.5cm^3$	2	2	2
Block, $5.0cm^3$	2	2	2
Block, $7.5cm^3$	2	2	2
Cricket ball	2	2	2
Sharpening stone	2	2	2
Pour water from one glass to another	2	1	1
Displace 2.25-cm alloy tube from one side of table to the other	2	2	2
Displace 1-cm alloy tube from one side of table to the other	1	1	2
